# Progress in the Management of Mediastinal Ectopic Parathyroid Adenomas: The Role of Minimally Invasive Surgery

**DOI:** 10.3390/jcm14093020

**Published:** 2025-04-27

**Authors:** Ioana-Medeea Titu, Cristina Alina Silaghi, Sergiu Adrian Ciulic, Florin Teterea, Monica Mlesnite, Emanuel Palade

**Affiliations:** 1 Department of Surgery, Iuliu Hatieganu University of Medicine and Pharmacy, 400000 Cluj-Napoca, Romania; titu_ioana_medeea@elearn.umfcluj.ro (I.-M.T.);; 2 Thoracic Surgery Clinic, Leon Daniello Clinical Hospital of Pneumology, 400371 Cluj-Napoca, Romania; 3 5th Department of Internal Medicine, Iuliu Hatieganu University of Medicine and Pharmacy, 400348 Cluj-Napoca, Romania; 4 Endocrinology Clinic, Cluj County Emergency Clinical Hospital, 400347 Cluj-Napoca, Romania

**Keywords:** primary hyperparathyroidism, mediastinal parathyroid adenoma, minimally invasive surgery, video-assisted thoracoscopic surgery (VATS), endocrine surgery

## Abstract

**Background/Objectives:** Primary hyperparathyroidism (PHPT) is a prevalent endocrine disorder, with ectopic mediastinal parathyroid adenomas accounting for up to 30% of cases, posing significant diagnostic and surgical challenges. While traditional management relies on invasive procedures, minimally invasive techniques such as video-assisted thoracoscopic surgery (VATS) have emerged as viable alternatives. This study addresses a gap in the current literature by presenting our experience with VATS for mediastinal ectopic parathyroid adenomas, particularly in underreported retrotracheal/paraesophageal locations. By integrating a retrospective case series with a systematic literature review, we highlight evolving surgical strategies and their implications for patient outcomes in anatomically complex cases. **Methods:** A retrospective analysis was conducted over a three-year period on patients diagnosed with mediastinal ectopic parathyroid adenomas. Data on demographic characteristics, preoperative imaging, surgical techniques, intraoperative findings, and postoperative outcomes were collected. This study primarily compared the outcomes of VATS with those of traditional thoracotomy, with a focus on surgical success, complication rates, and length of hospital stay. **Results:** Six patients underwent surgical resection for mediastinal ectopic parathyroid adenomas (two intrahymic and four retrotracheal/paraesophgeal). VATS was the preferred approach in all cases, with one patient requiring conversion to thoracotomy due to challenging vascular anatomy. Surgical success, defined as the normalization of postoperative serum calcium levels, was achieved in all cases. The median operative time was 80 min, and the mean hospital stay was 6.25 days. One patient developed transient postoperative hypocalcemia, necessitating supplementation. No major surgical complications were observed. **Conclusions:** This study supports VATS as a safe and effective approach for mediastinal ectopic parathyroid adenoma resection, offering reduced morbidity and shorter recovery times compared to traditional open surgery. The findings align with emerging evidence advocating for minimally invasive techniques in complex mediastinal surgeries.

## 1. Introduction

Primary hyperparathyroidism (PHPT) is a prevalent endocrine disorder characterized by elevated serum calcium and parathyroid hormone (PTH) levels. It is the third most common endocrine disorder after diabetes and thyroid disease, with a prevalence between 0.1–1% in the general population and an incidence of approximately 28 cases per 100,000 individuals annually [1,2]. Most cases are sporadic, with PHPT caused by a solitary parathyroid adenoma in 85–90% of patients, while multiglandular hyperplasia accounts for 10–15%, and parathyroid carcinoma is exceedingly rare (<1%) [1,2]. While parathyroid adenomas are predominantly located in the neck, 6–30% are ectopic, presenting significant diagnostic and surgical challenges [2,3,4].

Ectopic parathyroid adenomas arise due to aberrant embryological migration, predominantly affecting the inferior parathyroid glands, which share their developmental trajectory with the thymus from the third pharyngeal pouch [4,5]. The most common ectopic locations include the anterior mediastinum, retrotracheal/paraesophageal space, middle mediastinum, and carotid sheath [3,4,5]. Less frequent locations include the aortopulmonary window, pericardium, or diaphragm [2,3]. Identifying these atypical locations is essential to avoid surgical failure or reoperation [2,3].

Historically, the management of ectopic mediastinal parathyroid adenomas involved invasive procedures, such as thoracotomy or median sternotomy, which were associated with significant morbidity, prolonged recovery, and higher rates of complications [6]. However, advancements in imaging modalities, including high-resolution ultrasonography, SPECT/CT, and four-dimensional CT, have dramatically improved preoperative localization, enabling a more targeted surgical approach [2,6]. Radionuclide imaging with 99mTc-sestamibi, particularly in combination with SPECT/CT, remains a cornerstone for identifying ectopic parathyroid tissue, achieving a localization accuracy of up to 80% [7].

In recent decades, minimally invasive techniques, particularly video-assisted thoracoscopic surgery (VATS), have significantly improved the surgical management of ectopic mediastinal parathyroid adenomas [3,6]. VATS offers numerous advantages over traditional open surgery, including reduced postoperative pain, shorter hospital stays, faster recovery, and superior cosmetic outcomes [6,8]. Additionally, the minimally invasive approach provides excellent visualization of mediastinal structures, facilitates precise dissection, and reduces the risk of complications, achieving cure rates as high as 98–100% [6]. Intraoperative PTH monitoring further ensures complete excision of hyperfunctioning tissue, minimizing the likelihood of persistent or recurrent disease [2,9].

This article aims to present our experience with the minimally invasive VATS resection of mediastinal parathyroid adenomas. By detailing clinical presentations, preoperative imaging strategies, surgical techniques, and postoperative outcomes, we highlight the pivotal role of VATS in optimizing the management of ectopic parathyroid adenomas located in challenging mediastinal regions, addressing a significant knowledge gap in the current literature. While anterior mediastinal ectopic adenomas have been studied, retrotracheal/paraesophageal adenomas remain underevaluated. Furthermore, we emphasize the broader implications of minimally invasive approaches (such as VATS and prevertebral cervical techniques) in enhancing patient care and achieving superior outcomes for this rare but clinically significant condition. By integrating clinical data with a systematic review, this study provides insight into the evolving surgical strategies and their implications for patient follow-up.

## 2. Materials and Methods

### 2.1. Study Design and Data Collection

This research employs a dual methodology, comprising both a retrospective case series and a systematic literature review.

The retrospective analysis was conducted over a three-year period, evaluating patients diagnosed with mediastinal ectopic parathyroid adenomas who underwent surgical intervention. Data were collected from institutional medical records, including demographic details (age, sex), comorbidities, preoperative biochemical parameters, imaging findings, histopathological results, tumor size, prior parathyroidectomy history, surgical approach, postoperative complications, and length of hospital stay (LOS).

This retrospective case series aimed to describe the practical implementation and feasibility of minimally invasive surgical techniques, particularly VATS, in complex anatomical presentations such as retrotracheal/paraesophageal adenomas. Moreover, the study investigated the outcomes of different surgical approaches, including traditional thoracotomy, VATS/RATS, and cervical prevertebral. The choice of surgical technique was based on preoperative imaging localization and intraoperative findings.

### 2.2. Outcome Measures

The primary outcome was surgical success, defined as the normalization of postoperative serum calcium levels. Additional primary endpoints included complication rates, particularly postoperative hypocalcemia. The secondary outcomes included LOS and the need for additional surgical intervention.

### 2.3. Data Analysis

Data were systematically analyzed to compare the efficacy and safety of VATS versus open surgical techniques. Postoperative serum calcium levels were monitored at serial time points to assess recovery and resolution of hyperparathyroidism. Descriptive statistical analysis was performed to evaluate surgical success rates, postoperative morbidity, and hospital stay duration.

### 2.4. Surgical Technique

**Figure 1:**
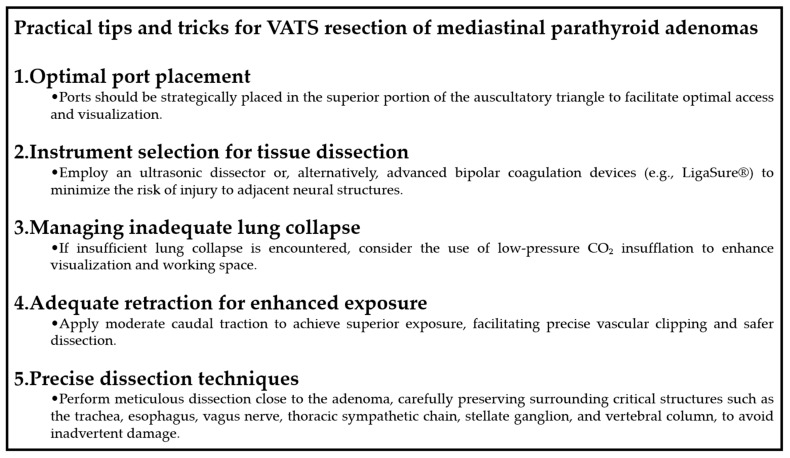
Practical tips and tricks for VATS resection of mediastinal parathyroid adenomas.

The surgical resection of an ectopic mediastinal parathyroid adenoma, particularly when located in the retrotracheal/paraesophageal region, requires a meticulous approach utilizing VATS to ensure precise resection while minimizing invasiveness. Figure 1 provides a summary of practical tips and technical recommendations aimed at optimizing outcomes and mitigating common intraoperative challenges during VATS resection of mediastinal parathyroid adenomas.

For this type of resection, general anesthesia with selective orotracheal intubation without CO_2_ insufflation is required, along with the possible placement of a nasogastric tube. The patient is positioned in a lateral decubitus position with the arm elevated and supported on a suspension frame.

The triportal VATS approach involves a 1.5–2 cm utility incision in the third intercostal space along the anterior axillary line, a 10 mm port for the video camera in the fourth intercostal space along the mid-axillary line, and, if necessary, an additional 5 mm port for surgical instruments in the fourth intercostal space along the posterior axillary line.

**Figure 2:**
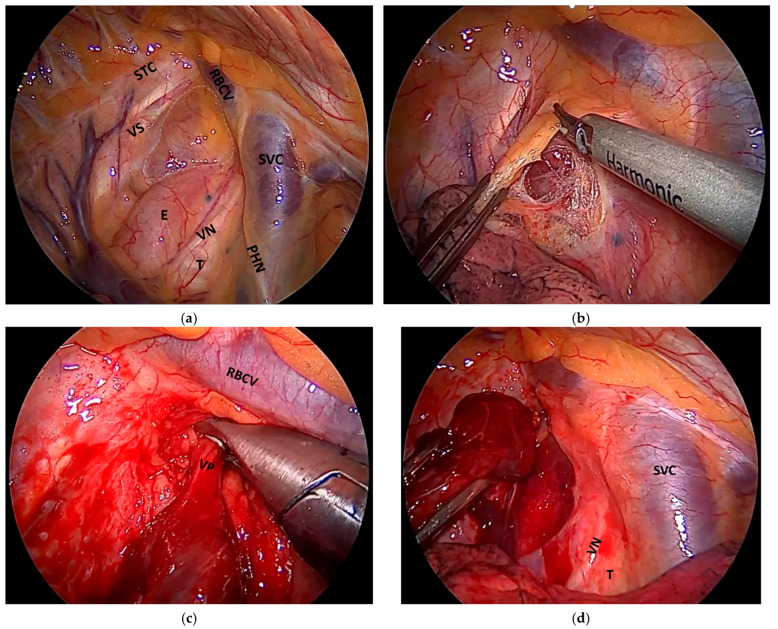
Surgical steps for VATS resection of an ectopic retrotracheal/paraesophageal parathyroid adenoma. Identification and intraoperative confirmation of the diagnosis (**a**), meticulous dissection of the adenoma using endoscopic instruments such as the harmonic scalpel to ensure precise tissue handling and minimal thermal damage (**b**), ligation of the vascular pedicle with titanium clips to control bleeding (**c**), and resection. After specimen retrieval, the pleural cavity is irrigated, and a pleural drain is placed (**d**). Acronyms: STC = sympathetic thoracic chain; VS = vertebral spine; RBCV = right brachiocephalic vein; SVC = superior vena cava; E = esophagus; VN = vagus nerve; T = trachea; PHN = phrenic nerve; VP = vascular pedicle of the adenoma.

Figure 2, along with the accompanying video (see Supplementary Materials), provides a detailed depiction of the surgical technique for the resection of a retrotracheal/paraesophageal adenoma.

The transpleural right-sided VATS thymectomy is our preferred technique for the excision of an intrathymic adenoma. This procedure requires general anesthesia with selective orotracheal intubation. For better exposure, low-pressure CO_2_ insufflation may be used. The patient is positioned in a lateral decubitus position, inclined at a 45-degree angle backward, with the arm elevated and supported on a suspension frame.

The right-sided transpleural VATS approach provides excellent visualization of the right phrenic nerve and the left brachiocephalic vein. The surgical approach involves a 1.5–2 cm utility incision in the third intercostal space along the anterior axillary line, a 10 mm port for the video camera in the fifth intercostal space along the anterior axillary line, and a 5 mm port for surgical instruments in the fifth intercostal space along the posterior axillary line.

The surgical procedure consists of the following steps: intraoperative identification and confirmation of the diagnosis, dissection using ultrasound-based surgical instruments (Harmonic scalpel), clipping of the thymic vascular pedicles, and extraction of the resected specimen using an endobag. Pleural drainage is established through the thoracoport incision used for the video camera.

### 2.5. Systematic Literature Review

To contextualize our findings, a systematic literature review was conducted, adhering to the Preferred Reporting Items for Systematic Reviews and Meta-Analyses (PRISMA) guidelines. The PubMed database was searched over a 10-year period using the keyword “mediastinal parathyroid adenoma”. The review encompassed all surgical approaches, including sternotomy, thoracotomy, robotic-assisted surgery, and VATS, with a focus on the advantages and outcomes of minimally invasive techniques.

Studies were included if they reported on patients with mediastinal ectopic parathyroid adenomas confirmed via imaging and histopathology and if they provided detailed information on surgical techniques, intraoperative and postoperative outcomes, and complications. Eligible studies included both prospective and retrospective analyses, published in English and available in full text.

**Figure 3:**
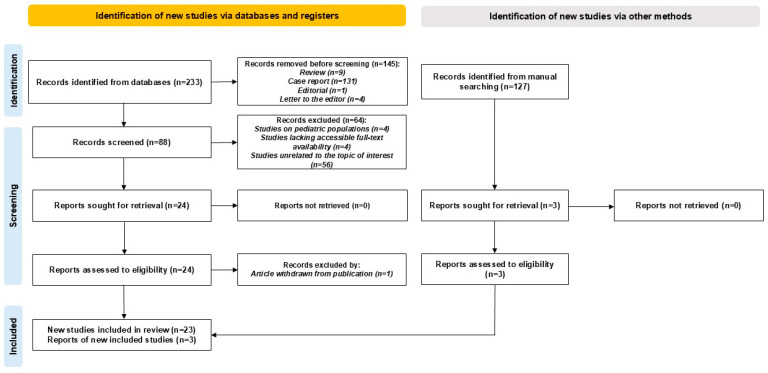
PRISMA diagram showing the results of the literature search.

Exclusion criteria comprised case reports, review articles, editorials, and letters to the editor, as well as studies focusing solely on pediatric populations or lacking accessible full texts. The study selection process is illustrated in Figure 3.

## 3. Results

Over a three-year period, at the Thoracic Surgery Clinic of Leon Daniello Clinical Hospital of Pneumology in Cluj-Napoca, six cases of mediastinal parathyroid adenomas were identified. Among these, two patients presented with intrathymic localization, while four had retrotracheal/paraesophageal ectopic parathyroid adenomas. This retrospective study focused on the minimally invasive surgical treatment of ectopic mediastinal parathyroid adenomas. In our cohort, all patients were diagnosed with PHPT confirmed through imaging and histopathological examination. The most frequently associated comorbidities included osteoporosis (*n* = 5, 83.3%), arterial hypertension (*n* = 3, 50%), and renal lithiasis (*n* = 2, 33.3%). Additionally, one patient had a history of lower limb varicose veins and another presented with hepatic steatosis. One patient had previously undergone a right nephrectomy due to nephrocalcinosis, and another had chronic renal insufficiency. Severe osteoporosis was reported in one case, while another patient had multiple comorbidities, including bronchial asthma and a history of breast cancer.

The histopathological examination confirmed ectopic mediastinal parathyroid adenomas in all four cases. The tumor volume, calculated using the ellipsoid formula (d_1_ × d_2_ × d_3_ × 0.52), ranged from 1.15 mL to 27.55 mL, yielding a total range of 26.40 mL, which demonstrates significant variability in tumor size across the studied cases.

VATS was the primary approach in all cases, with one patient requiring conversion to thoracotomy due to the deep-seated position of the adenoma’s vascular pedicle within the thoracic outlet, making it inaccessible through the VATS approach. The two patients with intrathymic adenomas underwent VATS thymectomy to ensure complete resection of the adenoma and to eliminate the possibility of smaller synchronous intrathymic adenomas. The operations were performed without any surgical complications. None of the patients had a history of prior parathyroidectomy. The median operative time was 80 min (range: 45–150 min). Postoperative nonsurgical complications occurred in one patient, who developed transient hypocalcemia. Table 1 presents the patients’ characteristics and summarizes the clinical evolution, treatment used, and outcome.

Surgical success was defined as the normalization of postoperative serum calcium levels within the normal range of 1.15–1.27 mmol/L. The mean preoperative serum calcium level was 1.57 mmol/L (range: 1.30–1.73 mmol/L). Postoperatively, all patients demonstrated a progressive decline in serum calcium levels, confirming the efficacy of surgical resection.

One patient developed postoperative hypocalcemia, requiring close monitoring and calcium supplementation.

The mean postoperative LOS was 6.25 days (range: 4–8 days). The patient who underwent thoracotomy had a LOS of 6 days, while patients treated with VATS had a mean LOS of 6.3 days. From a surgical perspective, the postoperative LOS was approximately two days, considering the removal of pleural drainage as the criteria for discharge. However, since all patients required monitoring and correction of their calcium levels, the actual LOS was notably prolonged.

**Figure 4:**
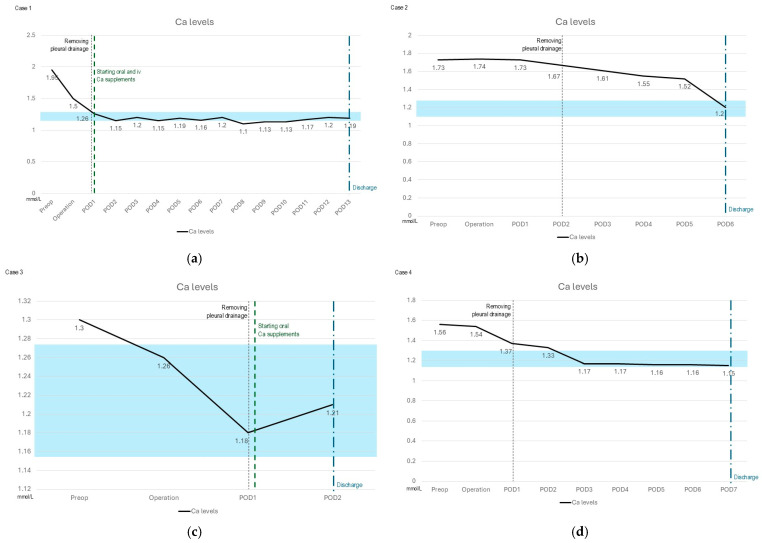

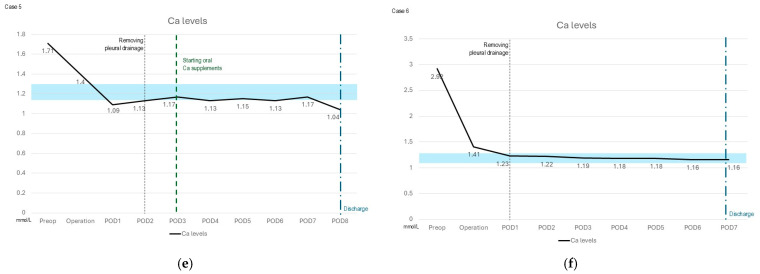
Postoperative course of serum calcium levels, calcium supplementation, chest drainage removal, and hospital discharge. The figure illustrates the temporal evolution of serum calcium levels, the administration of calcium supplementation, the postoperative day (POD) of chest drain removal, and the day of patient discharge (**a**–**f**). The shaded blue area in each graph denotes the reference range for normocalcemia (1.15–1.27 mmol/L).

Postoperative calcium levels were monitored at regular intervals. In all cases, there was an initial postoperative drop in calcium levels, followed by stabilization over subsequent days. Only one patient exhibited persistent hypocalcemia following hospital discharge, necessitating ongoing follow-up for treatment adjustment. Figure 4 presents the evolution of serum calcium levels, the administration of calcium supplementation (if required), the day of chest drainage removal, and the discharge day for the six cases analyzed.

## 4. Discussion

Ectopic mediastinal parathyroid adenomas pose significant diagnostic and therapeutic challenges due to their varied anatomical locations and complex surgical access. While anterior mediastinal adenomas are more commonly reported in the literature [10], our study presents a different distribution, with six cases—two intrathymic and four retrotracheal/paraesophageal—highlighting a higher proportion of adenomas in the posterior mediastinum. This distinction emphasizes the necessity of meticulous preoperative imaging and surgical planning, given that these adenomas frequently reside in proximity to important anatomical structures, including the esophagus, trachea, major blood vessels, and thoracic sympathetic trunk with stellate ganglia.

Traditionally, mediastinal parathyroid adenomas have been managed through open procedures such as sternotomy or thoracotomy, which offer excellent exposure and facilitate complete resection, particularly in cases involving large or deeply located tumors. Despite advancements in minimally invasive techniques, open surgery remains indispensable in complex cases where visualization and access are limited. Recent studies have further evaluated the role of open surgical approaches in optimizing patient outcomes and ensuring complete tumor excision, particularly in challenging anatomical locations.

Liu L. et al. conducted a 23-year study on ectopic mediastinal parathyroid tumors, analyzing 28 cases (35% of 80 diagnosed at their center). Of these, 26 were in the anterior superior mediastinum and 2 were in the middle mediastinum. While VATS was associated with shorter surgery time (*p* = 0.039) and less bleeding (*p* < 0.001), open surgery was necessary for cases with deep tumor locations (e.g., aortopulmonary window), large tumors (median 2 cm, range 1–8 cm), or prior failed neck explorations. In such cases, median sternotomy or thoracotomy was required [11]. The study by Du H. et al. (May 1995–May 2015) analyzed 21 patients (13 males, 8 females) with ectopic mediastinal parathyroid tumors. While 9 cases were treated with VATS, 13 required open surgery, including one converted from VATS. The study emphasized that tumors in the anterior mediastinum, near major vessels, or smaller than 1 cm were more challenging for thoracoscopic resection [12]. Their findings align with those of Liu et al. [11], confirming that while VATS is effective in selected cases, open surgery remains necessary when greater exposure is required.

Our study represents a significant advancement in the surgical management of mediastinal ectopic parathyroid adenomas, particularly those situated in anatomically challenging regions, such as a retrotracheal/paraesophageal location. In contrast to the broader yet more conventional 23-year retrospective review published by Liu L. et al. [11], which primarily reinforces established knowledge on anterior mediastinal lesions, the present study addresses a critical gap in the literature. Additionally, our cohort consisted entirely of female patients with a high prevalence of advanced comorbidities, such as osteoporosis (83.3%) and renal lithiasis (33.3%).

Considering the localization of ectopic mediastinal parathyroid adenomas, various minimally invasive approaches can be utilized, including VATS, robotic-assisted thoracic surgery (RATS), the prevertebral cervical approach, and the subxiphoid approach. The choice of technique depends on the precise anatomical positioning of the adenoma and its relation to surrounding structures.

While minimally invasive techniques have gained prominence, the decision between adenomectomy and thymectomy remains crucial in ensuring complete excision and preventing recurrence. Advances in surgical strategies, including VATS and the emerging subxiphoid approach, offer improved outcomes with reduced morbidity, particularly for lesions localized within the thymus or anterior mediastinum, being the most common ectopic mediastinal location of parathyroid ectopic tissue. When performing adenomectomy in the cervical region, if no ectopic adenomas are identified, surgical exploration can be extended through a cervical incision to assess for the presence of undetected adenomas in deeper mediastinal locations [3]. However, when preoperative imaging localizes the lesion within the thymus, VATS thymectomy is preferred to ensure complete excision and minimize recurrence risk. Our two cases of intrathymic parathyroid adenomas were managed using the VATS approach, aligning with the current literature pointing out minimally invasive strategies for these lesions. Both patients underwent successful resection without complications, supporting the advantages of VATS in reducing surgical morbidity, expediting recovery, and achieving a definitive biochemical cure.

A novel technique that has emerged in selected cases is the subxiphoid approach, which avoids intercostal nerve injury and reduces postoperative pain compared to traditional lateral thoracic access. This approach, widely utilized for thymectomy in the surgical management of myasthenia gravis, recently studied in a Japanese cohort, demonstrated superior cosmetic outcomes and comparable efficacy to VATS, although its use remains limited to specific mediastinal locations, being primarily suitable for lesions located in the thymic region (intrathymic adenomas), the prevascular space, the retrosternal and pericardial areas, as well as the para-aortic area and aortopulmonary window [13].

While anterior mediastinal adenomas are often accessible via a transcervical approach, deeper-seated lesions necessitate minimally invasive thoracoscopic techniques such as VATS or, in some cases, RATS [13]. Adenomas located in the visceral mediastinum, particularly retrotracheal/paraesophageal parathyroid adenomas, require a careful selection of minimally invasive approaches, including VATS, RATS, and the prevertebral cervical approach. These methods replace open surgery, thereby reducing morbidity and enhancing recovery. VATS is one of the most frequently employed techniques, as it enables precise dissection and direct visibility within the mediastinum. This procedure is particularly advantageous for adenomas located beneath the thoracic outlet, where a transcervical approach is ineffective. It enables the precise identification and ligation of feeding vessels prior to deep mediastinal dissection, thereby reducing the risk of intraoperative hemorrhage and preserving the adjacent neurovascular structures. VATS reduces pain and hospitalization by employing an endoscopic camera and small thoracic incisions to reveal the lesion [12].

**Figure 5:**
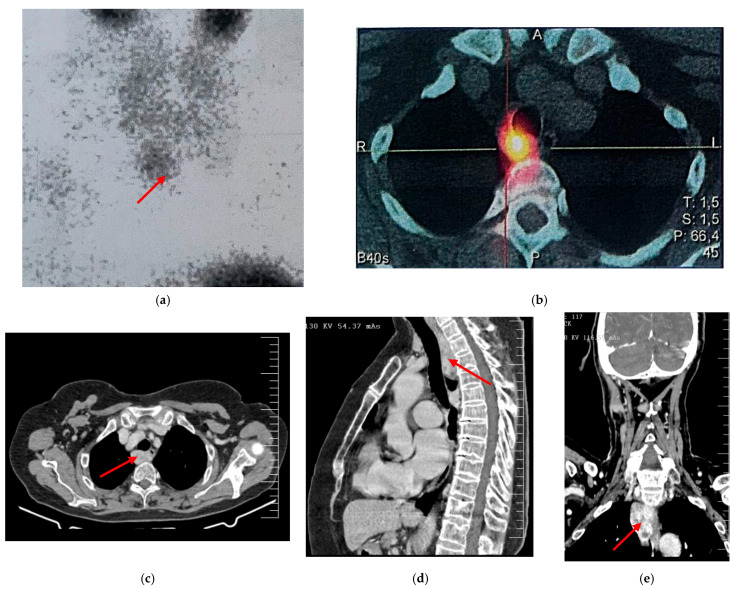
Preoperative imaging of a retrotracheal/paraesophageal adenoma (pointed with a red arrow), including contrast-enhanced CT and sestamibi scintigraphy. Parathyroid scintigraphy (**a**) performed using the “washout” technique revealed: radiopharmaceutical uptake at the upper mediastinal level. This area persists with increased metabolic activity in the late images. SPECT/CT acquisition (**b**) highlights an uptake area corresponding to a structure located retrotracheally, right paraesophageal, slightly shifting the esophagus to the left, reaching the level of the D4 vertebral body, measuring 19 mm, suggesting a hyperfunctioning ectopic parathyroid gland. There is no other pathological MIBI uptake at the cervical or ectopic level. CT scans (**c**–**e**) show the retrotracheal/paraesophageal adenoma.

Our study is consistent with these findings, reporting four cases of retrotracheal/paraesophageal parathyroid adenomas requiring VATS resection, one of which necessitated conversion to thoracotomy. Among the cases managed exclusively with VATS, the median operative time was 50 min, while the length of hospital stay ranged from 4 to 8 days, with a median of 7 days. Figure 5 illustrates the preoperative imaging of a retrotracheal/paraesophagealadenoma, including contrast-enhanced CT and sestamibi scintigraphy.

The success rate of VATS in our series aligns with previous reports, with all patients achieving postoperative normocalcemia. Our findings are comparable to those of Wang et al. (2022) and Ramonell et al. (2022), who reported cure rates approaching 100% following minimally invasive resection [7,14]. However, in contrast to Wang et al., who observed postoperative hypocalcemia in 83.3% of cases, our incidence was significantly lower at 16.7%, suggesting potential benefits from more rigorous perioperative calcium monitoring and supplementation [7].

In terms of patient characteristics, our cohort exhibited a higher prevalence of osteoporosis (83.3%) and arterial hypertension (50%), suggesting a more advanced disease stage at diagnosis. The frequent association with PHPT-associated renal comorbidities further indicates a complex clinical profile that may influence both surgical outcomes and postoperative management [15]. While surgical recovery was generally rapid, hospitalization was primarily prolonged due to calcium monitoring rather than surgical morbidity [16]. Unlike their cervical counterparts, mediastinal ectopic parathyroid adenomas may remain asymptomatic for prolonged periods [17].

**Figure 6:**
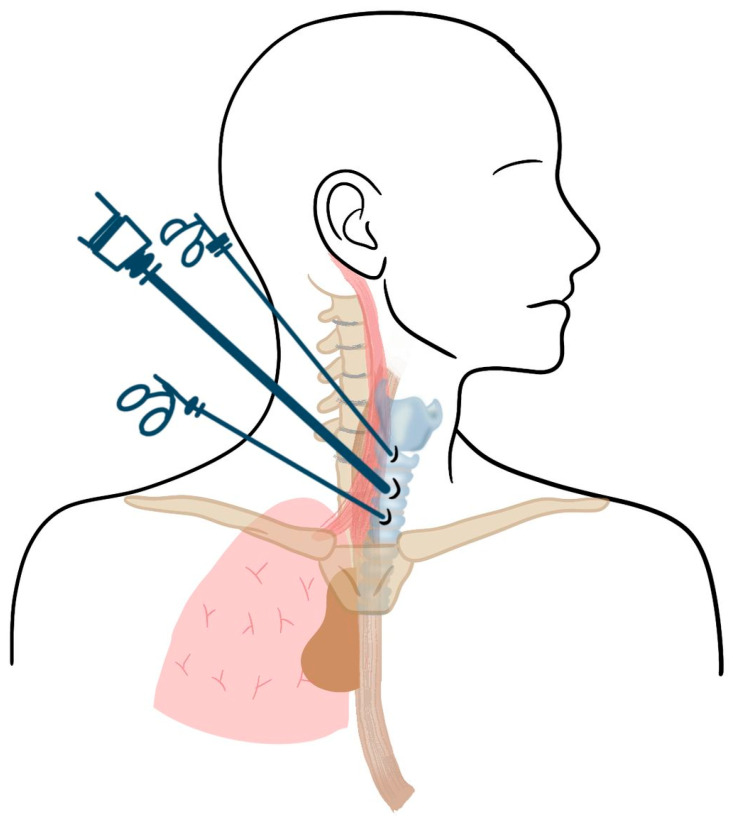
Patient positioning and trocar placement for prevertebral cervical surgical access.

An emerging alternative to VATS for retrotracheal/paraesophageal adenomas is the prevertebral cervical approach, first described by Martos-Martínez et al. (2017) and later expanded upon by Rubio-Manzanares Dorado et al. (2022). This approach offers a pure endoscopic cervical technique for posterior mediastinal adenomas, eliminating the need for thoracic incisions and single-lung ventilation [18,19]. The prevertebral cervical approach is a minimally invasive technique for removing posterior mediastinal parathyroid adenomas without pleural entry. It begins with patient positioning in slight neck hyperextension, followed by a 1 cm incision along the anterior border of the sternocleidomastoid muscle. After blunt dissection to expose the prevertebral fascia, CO_2_ insufflation creates a working space for three trocars: an 11 mm trocar for optics and extraction and two 5 mm trocars (or one 5 mm and another 2.8 mm trocar) for instrumentation (Figure 6). Dissection is performed using fine-tipped bipolar energy devices (e.g., the 5 mm, 20 cm LigaSure^®^ dolphin tip instrument) or ultrasonic devices (e.g., Harmonic™ scalpel), ensuring minimal risk to adjacent structures. A suction–irrigation system is essential for maintaining a clear operative field, while hemostatic agents (e.g., Surgicel™) help control intraoperative bleeding, particularly in hypervascular adenomas. The technique is optimal for adenomas between 1–4 cm in diameter, as smaller lesions may be difficult to locate, and larger ones may be challenging to manipulate in the confined space [18].

Rubio-Manzanares Dorado M. et al. conducted a retrospective study of a prospectively maintained database, evaluating the outcomes of ten patients who underwent a prevertebral cervical approach for posterior mediastinum parathyroid adenomas between June 2015 and January 2021. The mean operative time was 33 ± 7 min, with no intraoperative complications or conversions to open surgery. Postoperatively, seven patients experienced mild subcutaneous emphysema, and one patient had a transient recurrent laryngeal nerve injury, which resolved within a month. All patients were discharged within 1–3 days, with normalized calcium and parathyroid hormone levels, except for one case of permanent hypoparathyroidism due to previous total parathyroidectomy. The findings suggest that this approach is a safe, effective, and minimally invasive alternative to traditional thoracic procedures, offering short hospital stays, low complication rates, and excellent cosmetic outcomes [19].

Despite these advantages, the prevertebral cervical approach has significant limitations. Unlike VATS, which provides direct visualization of the entire posterior mediastinum, the prevertebral cervical approach is constrained to adenomas located between the D2 and D5 vertebral levels, making it unsuitable for deeply intrathoracic lesions or those extending into the lower mediastinum. Furthermore, VATS allows for direct exposure and precise dissection, which may be difficult to access via the prevertebral approach due to the confined surgical field. The requirement for CO_2_ insufflation in the cervical and prevertebral regions may also pose additional risks for patients. Another limitation of the prevertebral cervical approach is the requirement for specialized instruments adapted to the restricted working space. Unlike VATS, which utilizes standard thoracoscopic instruments, the prevertebral technique necessitates the use of shorter instruments (20 cm) to facilitate maneuverability within the narrow prevertebral plane [18].

Considering the advantages and disadvantages inherent to each technique, the VATS and prevertebral cervical approaches for retrotracheal/paraesophageal parathyroid adenomas can be considered complementary surgical techniques. The alternative approach is a viable conversion strategy that ensures effective resection while minimizing the need for more invasive procedures in cases where lesion excision is not feasible using one method. To optimize this strategy, patient positioning on the operating table should allow for a seamless transition between approaches without requiring repositioning. A semi-supine position with slight neck extension facilitates both cervical access and thoracoscopic maneuvers. Both VATS and prevertebral cervical approaches require minimally invasive tools for safe dissection, hemostasis, and tumor removal. However, the prevertebral cervical approach necessitates shorter instruments due to the confined surgical field, requiring a dedicated instrument tray.

The choice of the initial approach depends on key anatomical and radiological criteria: for adenomas located in the upper posterior mediastinum or near the thoracic inlet, a cervical approach is prioritized, while deep-seated or lower mediastinal lesions are better approached via VATS. In cases of uncertain localization or intraoperative difficulties, early conversion between techniques can enhance surgical safety, ensuring complete resection while minimizing morbidity. Table 2 proposes a minimally invasive approach according to the ectopic location of mediastinal parathyroid adenomas, while also outlining the alternative conversion strategy when necessary.

Mediastinal parathyroid adenomas located in the aortopulmonary window, pericardium, and diaphragm require an adapted surgical approach depending on each localization. For aortopulmonary window adenomas, a left-sided VATS approach provides optimal exposure, while a right-sided approach may be required for lesions extending in the right paratracheal region [6]. Surgical approaches for pericardial parathyroid adenomas depend on their location and complexity, with minimally invasive techniques such as VATS and RATS being suitable for small, anteriorly located adenomas, though they are limited in accessing deeply embedded or posterior lesions. Open surgical approaches are necessary for more complex cases, with median sternotomy preferred for central adenomas near major blood vessels, thoracotomy used for laterally positioned adenomas, and a transcervical approach considered for superiorly located lesions, though it may require conversion to a more invasive technique. Minimally invasive approaches, such as VATS, RATS, and laparoscopic transabdominal surgery, are preferred for superficial diaphragmatic adenomas, providing precise access while minimizing surgical trauma, though they are less effective for deep or posterior lesions. In contrast, open surgical approaches like thoracotomy, median sternotomy, and laparotomy are required for deeply embedded, large, or complex adenomas, ensuring direct visualization and complete excision, often necessitating multidisciplinary collaboration [3].

The application of robotic-assisted techniques in mediastinal parathyroidectomy is gaining traction, particularly for challenging or reoperative cases. Ramonell et al. (2022) reported the largest case series (16 patients) on robotic-assisted transthoracic mediastinal parathyroidectomy (TTRMP), demonstrating its safety and effectiveness, particularly for deep-seated adenomas inaccessible via a transcervical approach. However, complications such as transient recurrent laryngeal nerve palsy and deep vein thrombosis were noted [14]. Compared to robotic techniques, VATS in our study demonstrated similar success rates while avoiding the increased operative time and resource utilization associated with robotic surgery. While robotic techniques provide enhanced maneuverability and 3D visualization, their high cost and limited availability remain barriers to widespread adoption.

As minimally invasive approaches such as VATS have become the preferred technique for the resection of ectopic mediastinal parathyroid adenomas, their effectiveness can be limited in cases where adenomas are deeply embedded or difficult to visualize intraoperatively. According to a study comparing VATS and open surgery, adenomas measuring less than 1 cm present a particular challenge, often requiring additional localization techniques such as intraoperative near-infrared (NIR) fluorescence imaging or methylene blue staining to improve detection rates [12,20]. Other intraoperative techniques have also been explored to enhance localization accuracy. Radioguided surgery using low-dose technetium-99m (Tc-99m) sestamibi has demonstrated high sensitivity in detecting ectopic mediastinal parathyroid adenomas, particularly in cases where preoperative imaging is inconclusive [21]. Additionally, indigo carmine injection has been proposed as an alternative dye-based method for intraoperative identification of ectopic adenomas. In a preliminary study, this technique improved the visualization of adenomas, allowing for more precise excision, particularly in patients with challenging mediastinal locations [22].

The intraoperative parathyroid hormone (ioPTH) assay is widely used to confirm the complete removal of hyperfunctioning parathyroid tissue by assessing a significant postoperative PTH decline of at least 50% from baseline within minutes of gland excision, reducing the risk of reoperation and persistent disease. While particularly beneficial in cases of uncertain preoperative localization or suspected multiglandular disease, its necessity remains debated [23]. This was not applicable in our study, as the lesions were readily identified intraoperatively, as demonstrated by intraoperative imaging and the tumor volumes documented in the tables. Studies suggest that experienced surgeons can achieve similar success rates without ioPTH when preoperative imaging reliably identifies a solitary adenoma. Previously used methods, such as intraoperative frozen section analysis and contralateral gland biopsy, have largely been abandoned due to their limitations. Ultimately, while ioPTH remains a valuable tool, it may not be essential in well-selected cases with precise preoperative localization and no suspicion of multiglandular disease [24].

Our study corroborates findings from previous research supporting the advantages of VATS. Like the case series by Wang et al. (2022) [7], which evaluated six patients undergoing minimally invasive surgery for mediastinal adenomas, we observed rapid postoperative normalization of calcium levels in all cases. However, Wang et al. highlighted a higher incidence of postoperative hypocalcemia (83.3%) compared to our study (16.7%), emphasizing the need for vigilant calcium monitoring and supplementation following VATS resections [24].

A key limitation of this study is its retrospective design and small sample size (*n* = 6), which restricts statistical power and precludes the definitive validation of prior findings. Nonetheless, our institutional experience is rooted exclusively in minimally invasive approaches (VATS), reflecting a broader paradigm shift in thoracic and endocrine surgery. Consequently, no internal comparator group undergoing traditional thoracotomy or sternotomy is available. However, this absence should not detract from the validity of our findings. VATS has emerged as the standard of care for technically resectable mediastinal parathyroid adenomas, mirroring similar shifts seen in other surgical fields, such as the replacement of open surgery by laparoscopic cholecystectomy. As with these transitions, the widespread clinical adoption of VATS is underpinned by outcome-based evidence rather than randomized controlled trials, which may be unnecessary or ethically impractical when one technique clearly offers superior recovery, reduced morbidity, and equivalent or improved efficacy. Our systematic review further reinforces this position, as the published literature consistently supports the safety and effectiveness of VATS over open approaches. Thus, while our small cohort limits generalizability, the convergence of institutional data and cumulative external evidence underscores the appropriateness and clinical superiority of the minimally invasive strategy employed.

Additionally, this study provides new insights into the evolving field of minimally invasive surgery for mediastinal ectopic parathyroid adenomas, with a particular emphasis on retrotracheal/paraesophageal localizations, which are currently underrepresented in the literature. Our results further support the feasibility and efficacy of VATS as a primary surgical approach, offering optimal exposure, high cures rates, and very low morbidity. We recommend conversion between the VATS and prevertebral cervical approaches; however, this necessitates preoperative planning, including patient positioning on the operating table and the availability of appropriate surgical instruments.

Furthermore, we highlight the clinical importance of perioperative calcium monitoring, which continues to be a critical factor in postoperative recovery. We offer a thorough assessment of minimally invasive strategies, emphasizing their ability to improve patient outcomes and mitigate surgical trauma, by combining our institutional experience with a systematic literature review.

## 5. Conclusions

By integrating a detailed clinical case analysis with a systematic review, our study not only highlights the efficacy of VATS in underreported and anatomically challenging settings, such as retrotracheal/paraesophageal locations, but also proposes a refined, localization-based surgical algorithm.

This anatomically adapted approach reinforces the role of VATS as a safe and effective technique for mediastinal parathyroid adenomas, particularly in retrotracheal/paraesophageal locations, offering significant advantages in terms of patient recovery, postoperative pain, and reduced postoperative complications, compared to open surgery (thoracothomy/sternothomy), with comparable outcomes to other minimally invasive approaches.

Nevertheless, the results are consistent with a growing body of literature that supports minimally invasive techniques as the preferred approach in appropriately selected cases. The advantages of VATS are well-documented and have increasingly become standard practice in thoracic and endocrine surgery. Given this trajectory, large randomized controlled trials may be impractical or ethically unjustified, particularly when open surgery is associated with increased patient risk and morbidity.

The prevertebral cervical and subxiphoidian approaches emerge as promising alternatives to VATS or RATS for selected cases. Robotic-assisted surgery remains an advanced technique suitable for challenging cases, albeit with higher costs and longer operative times. The development of intraoperative visualization techniques such as NIR fluorescence imaging or methylene blue staining, radioguided surgery using low-dose technetium-99m (Tc-99m) sestamibi, and indigo carmine injection may further improve surgical management and expand the role of minimally invasive surgery in mediastinal parathyroidectomy.

However, further multicentric, prospective investigations are necessary to validate these findings, expand the evidence base, and refine the selection criteria for alternative minimally invasive strategies such as the prevertebral cervical or subxiphoid approaches.

## Figures and Tables

**Table 1:** Characteristics of the patients, treatment used, evolution and outcome.

	Tumor Localization	Sex	Age	Type of HPT	Comorbidities	Histology	Size (cm)	Volume (mL)	Previous Parathyroidectomy	Surgical Approach	Surgery Duration (min)	Complications	Postoperative LOS (days)
Case 1	Intrathymic	F	58	PHPT	Osteoporosis, gout	Ectopic mediastinal parathyroid adenoma	1.5/1.4/2.2	2.40	No	VATS thymectomy	100	No	13
Case 2	Retrotracheal/ paraesophageal	F	61	PHPT	Secondary vertebral osteoporosis, renal lithiasis, chronic renal insufficiency, non-toxic multinodular goiter, arterial hypertension	Ectopic mediastinal parathyroid adenoma	3.5/3/2	10.92	No	VATS, conversion to thoracotomy	150	No	6
Case 3	Retrotracheal/ paraesophageal	F	48	PHPT	Right nephrectomy, arterial hypertension, hypercholesterolemia, lower limb varicose veins	Ectopic mediastinal parathyroid adenoma	3/2.1/0.8	2.62	No	VATS	50	No	4
Case 4	Retrotracheal/ paraesophageal	F	69	PHPT	Severe osteoporosis, renal lithiasis, arterial hypertension, lower limb varicose veins, hepatic steatosis	Ectopic mediastinal parathyroid adenoma	4.8/4.8/2.3	27.55	No	VATS	60	No	7
Case 5	Retrotracheal/ paraesophageal	F	72	PHPT	Osteoporosis, renal lithiasis, bilateral profound sensorineural hearing loss, coxarthrosis gonarthrosis	Ectopic mediastinal parathyroid adenoma	5.5/3/2	17.16	No	VATS	45	Hypocalcemia	8
Case 6	Intrathymic	F	73	PHPT	Osteoporosis, arterial hypertension, left breast cancer, MRM, hormone-treated, bronchial asthma	Ectopic mediastinal parathyroid adenoma	2/1/1.1	1.15	No	VATS thymectomy	80	No	7

**Table 2:** Proposal of minimally invasive approaches for ectopic mediastinal parathyroid adenomas based on the literature and our experience.

Localization	Preferred Approach	Alternative Approach
Anterior mediastinum (intrathymic)	VATS/RATS	Subxiphoid Cervical thymectomy
Retrotracheal/paraesophageal with high seated vascular pedicle (D1–D4)	Prevertebral cervical	VATS/RATS
Retrotracheal/paraesophageal with low-seated vascular pedicle (D5–D8)	VATS/RATS	Prevertebral cervical
Aortopulmonary window	VATS/RATS	-
Pericardial	VATS/RATS	Subxiphoid
Diaphragm	VATS/RATS	Laparoscopic transabdominal

## Data Availability

The data supporting the findings of this study are available from the corresponding author upon reasonable request. Due to ethical considerations, the data is not publicly accessible.

## Supplementary Materials

The following supporting information can be downloaded at: https://www.mdpi.com/article/10.3390/jcm14093020/s1.
